# Dysregulated Neurotransmission induces Trans-synaptic degeneration in reconstructed Neuronal Networks

**DOI:** 10.1038/s41598-018-29918-1

**Published:** 2018-08-02

**Authors:** Bérangère Deleglise, Benjamin Lassus, Vanessa Soubeyre, Mohamed Doulazmi, Bernard Brugg, Peter Vanhoutte, Jean-Michel Peyrin

**Affiliations:** 1 CNRS UMR 8256, Biological Adaptation and Ageing, Paris, F-75005 France; 2 Sorbonne Universités, UPMC Université Paris 06, CNRS/UMR 8256, Institut de Biologie Paris Seine, Paris, F-75005 France; 3 Sorbonne Universités, UPMC Université Paris 06, Neurosciences Paris Seine, CNRS/UMR8246-INSERM/UMR-S1130, Institut de Biologie Paris Seine, Paris, F-75005 France

## Abstract

Increasing evidence suggests that pathological hallmarks of chronic degenerative syndromes progressively spread among interconnected brain areas in a disease-specific stereotyped pattern. Functional brain imaging from patients affected by various neurological syndromes such as traumatic brain injury and stroke indicates that the progression of such diseases follows functional connections, rather than simply spreading to structurally adjacent areas. Indeed, initial damage to a given brain area was shown to disrupt the communication in related brain networks. Using cortico-striatal neuronal networks reconstructed in a microfluidic environment, we investigated the role of glutamate signaling in activity-dependent neuronal survival and trans-synaptic degeneration processes. Using a variety of neuronal insults applied on cortical neurons, we demonstrate that acute injuries such as axonal trauma, focal ischemia, or alteration of neuronal rhythms, lead to glutamate-dependent striatal neuron dysfunction. Interestingly, focal pro-oxidant insults or chronic alteration of spontaneous cortical rhythms provoked dysfunction of distant striatal neurons through abnormal glutamate GluN2B-NMDAR-mediated signaling at cortico-striatal synapses. These results indicate that focal alteration of cortical functions can initiate spreading of dysfunction along neuronal pathways in the brain, reminiscent of diaschisis-like processes.

## Introduction

Diaschisis is defined as a dysfunction in an area of the brain connected to a distant, damaged, brain area^1^. The primary mechanisms of diaschisis are functional and structural deafferentiation that lead to loss of input information from the damaged brain area. This is followed at later stages by reorganization of distributed brain networks in the distantly targeted area^2–4^. Diaschisis has mostly been described after focal stroke or traumatic brain injuries, however, recent evidence from neurodegenerative syndromes shows that trans-neuronal degeneration also occurs in Alzheimer’s or Parkinson’s diseases^5^, suggesting that trans-synaptic dysfunction is an event common to a variety of brain pathologies.

While functional deafferentiation is thought to be one of the primary mechanisms of diaschisis, the cellular and molecular mechanisms involved in trans-synaptic degeneration processes remain unknown. The control of glutamate release/recapture shapes normal brain functions, and alterations in glutamate neurotransmission are strongly associated with both acute and chronic degenerative processes (see review^6^). Indeed, glutamate, the most abundant excitatory neurotransmitter in the central nervous system, plays a key role in controlling neuronal activity-dependent survival pathways^7^ that lead to the expression of pro-survival genes, increase of anti-oxidative defense factors, and inhibition of pro-apoptotic molecules. On the other hand, overstimulation of NMDA receptors (NMDAR) by excessive glutamate levels has long been associated with excitotoxicity-induced neuronal death through calcium increase, energetic imbalance, and activation of death associated pathways; possibly through extra-synaptic NMDAR activation^7,8^. Glutamate therefore acts as a hub controlling neuronal network robustness under stress^9–11^. While these processes are under active investigation, the consequences of locally initiated synaptic/extra-synaptic stimulations on distant, unchallenged post-synaptic neurons are not known. Dysfunctions of glutamatergic neurotransmission have been associated with primary and secondary progression of degeneration following acute lesions (e.g. epilepsy, ischemia, and traumatic brain injuries)^12–14^. A similar scenario has been proposed in neurodegenerative syndromes such as amyotrophic lateral sclerosis, Huntington disease, Parkinson’s or Alzheimer’s diseases^15–19^. Classically, the molecular mechanisms of trans-synaptic degeneration processes have been studied using organotypic brain slices and axonal trauma paradigms^20,21^; however, using these methods, unravelling the molecular consequences of focal and chronic insults on distant connected brain areas remains a challenging issue, and calls for the development of new experimental tools.

Here, using *in vitro* reconstructed, oriented cortico-striatal networks in microfluidic chambers^22^, we modeled alterations of glutamatergic activity in cortical neurons in order to analyze their impact on survival of post-synaptic striatal neurons. Corroborating previous work on brain slices, we found that axotomy of cortical fibers induced trans-synaptic striatal neuron degeneration that was mainly due to NMDAR-mediated excitotoxicity, rather than “passive” loss of presynaptic input. Indeed, chronic cortical stresses, such as ischemia or aberrant stimulation of cortical NMDAR, led to an important trans-synaptic degeneration process in connected striatal neurons that involved postsynaptic striatal GluN2B-containing NMDARs. These results show that glutamate-dependent trans-synaptic dysfunction in distant neurons is not only triggered by physical lesions of cortical axons, but can also be initiated by focal chronic injuries. Interestingly, our results, which are reminiscent of diaschisis-like processes, also indicate that chronic alteration of neuronal rhythmic patterns can initiate the spreading of neuronal degeneration processes along a neuronal network.

## Results

### Blockade of cortical activity does not induce trans-synaptic degeneration

**Figure 1:**
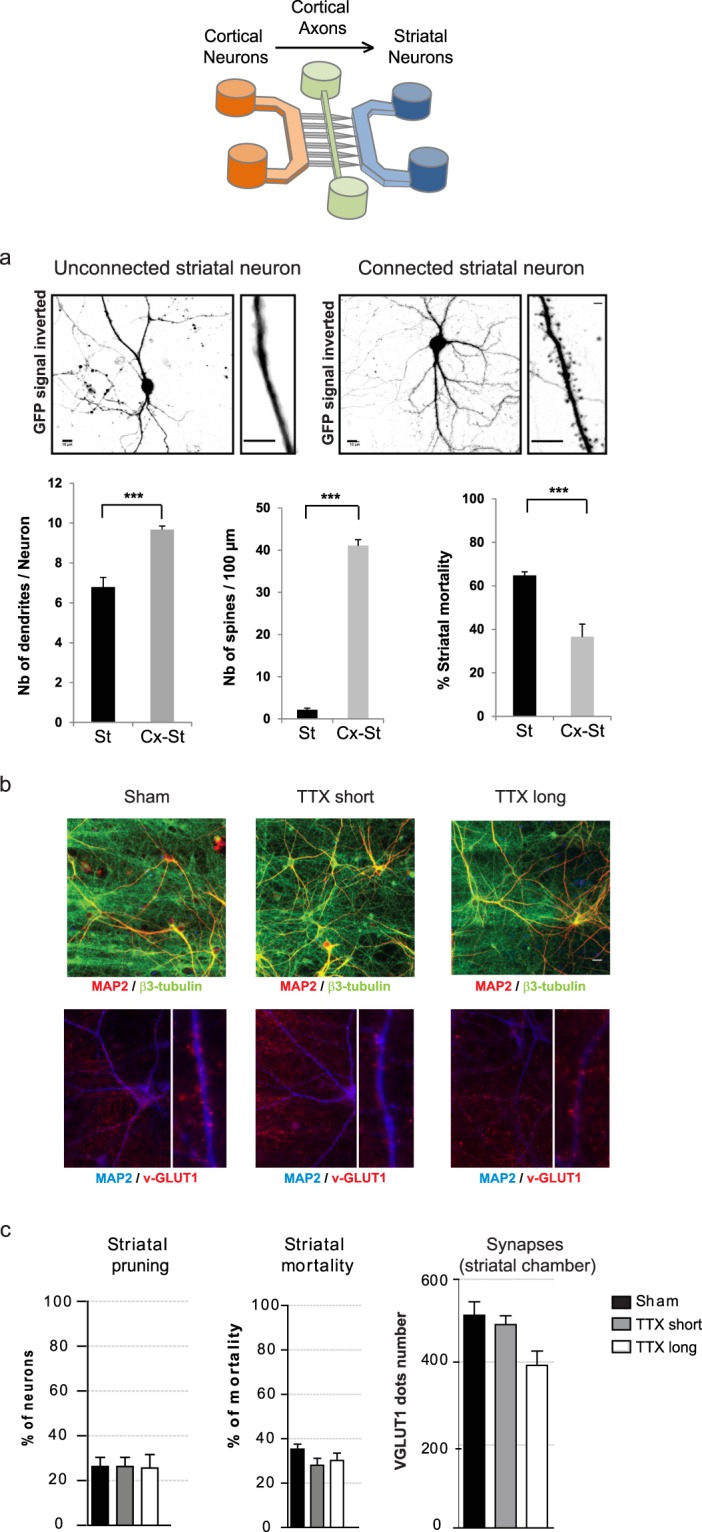
Paralysis of cortical neurons does not induce trans-synaptic degeneration. Microfluidic neuronal culture devices are composed of two separate cell culture chambers (proximal and distal) interconnected by a series of asymmetrical micro-channels allowing passage of axons only in left-to-right direction (cortical axons towards striatal neurons). A central narrow channel (central) gives access to the central part of axons. Micro-channels. (**a**) 15 DIV striatal neurons grown in microfluidic chambers without (left panels), and with cortical afferences (right panels). Striatal neurons were infected with a GFP expressing lentivirus in order to evidence dendritic spines along striatal dendrites (inserts). Below: Quantification at 15 DIV of the number of dendrites per neuron, number of dendritic spines per 100 µm of dendritic length, and striatal neuronal survival. Scale bar 10 µM. (**b**) Cortical neuronal activity was blocked with 1μM of tetrodotoxin for 10 min (TTX short), or 24 hours (TTX long), without treating the receiving chamber containing cortical axon endings and striatal neurons. Both cortical axons (α-tubulin in green) and striatal dendrites (MAP2 in red) remained unaffected. However, a slight but not significant loss of presynaptic proteins was observed upon long cortical activity blockade (lower panels, cortical VGLUT1 in red, MAP2 in blue, insert are magnification of striatal dendritic segments). Scale bar 10 µm. (**c**) Quantification of striatal pruning, striatal mortality, and VGLUT1 positive structures along striatal dendrites after sham, short, or long TTX treatment.

We previously showed that growing cortical and striatal neurons in “axon diode” microfluidic chips allows for the reconstruction of oriented and functional neuronal networks *in vitro*^22^. Cortical neurons seeded in the “emitting chambers” typically project their axons toward the striatal “receiving chamber” after 7 days *in vitro* (DIV). As shown in Fig. 1a, 15 days after seeding, cortical innervation triggers the appearance of spines on striatal dendrites, leading to a higher dendritic complexity compared to striatal neurons cultured alone (Fig. 1a). Due to low neuronal density at initial seeding, a proportion of striatal neurons spontaneously degenerated during the first week of culture. Interestingly, the progressive cortical connection is accompanied by an increase in striatal neuron survival, with the spontaneous death rate dropping from 65% when striatal neurons are grown by themselves, to 40% upon connection to cortical axons. As this rescue phenomenon is possibly linked to synaptic activity, we wondered whether in turn, blockade of cortical activity would trigger spontaneous degeneration of striatal target neurons. To this end, the emitting chamber was incubated with 1μM of tetrodotoxin for 10 min (TTX short) or 24 hours (TTX long), without treating the receiving chamber containing cortical endings and striatal neurons. Both cortical axon terminals (revealed with α-tubulin in green) and striatal dendrites (revealed with MAP2 in red) remained unaffected by short and long TTX treatments (Fig. 1b,c). Staining of the striatal compartment with VGLUT1 antibody reveals glutamatergic vesicles present in cortical neurons. While VGLUT1 vesicles can be observed in growing cortical axons, VGLUT1 positive structures progressively cluster next to the striatal dendrites between DIV 12 and DIV 15 (see Fig. 1b Sham insert and our previous studies^22,23^). We therefore consider these VGLUT1 vesicle clusters on MAP2 striatal dendrites as a proxy of cortico-striatal connectivity. Here, although a tendency of lower presynaptic protein levels (cortical VGLUT1 clusters in red, apposed to MAP positive striatal dendrites in blue, Fig. 1b,c) was observed upon long-lasting cortical activity blockade, it was not significant. This suggests that long-term loss of cortical activity does not trigger significant spontaneous degeneration or regression of striatal neurons in our model.

### Cortical axotomy or inhibition of glutamate reuptake by cortical axons triggers NMDA-Receptor-mediated trans-synaptic degeneration of striatal neurons

**Figure 2:**
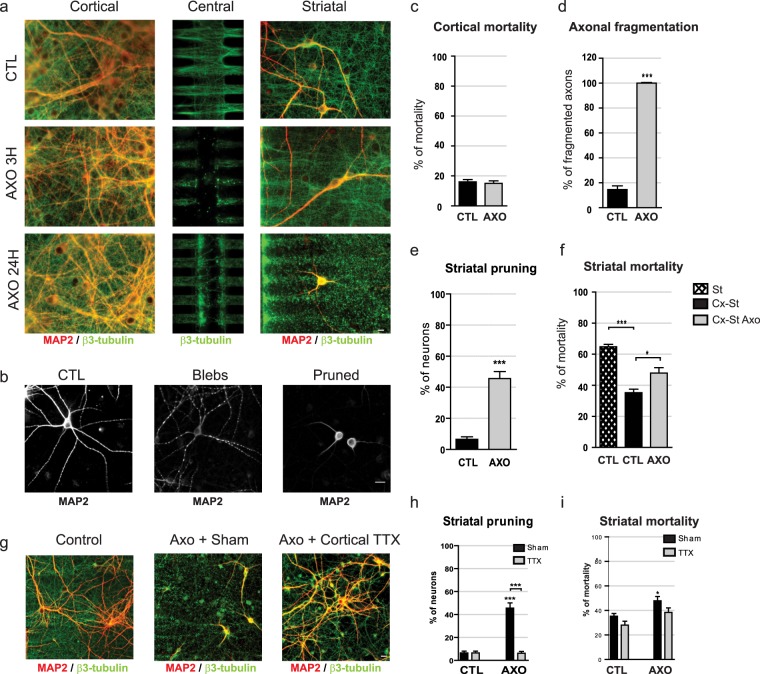
Cortical axotomy induces trans-synaptic degeneration in striatal neurons. (**a**) Cortical, central, and striatal chambers of normal (CTL) or axotomized (AXO3h, AXO24h) reconstructed cortico-striatal networks. Neurons were stained for MAP-2 (red), α-tubulin (green). Cortical neurons send axons efficiently through the micro-channels and the central chamber to reach, and connect with, striatal neurons. While cortical axons can be evidenced in the central chamber of CTL networks, 3 hours after axotomy the central chamber is void of axons, but axons inside the micro-channels and in the striatal chamber remain intact. Twenty-four hours after cortical axotomy all cortical axons disconnected from their soma are fragmented, and both the survival and the complexity of the dendritic arborization of remaining striatal neurons were altered (MAP-2, red) (Fig. 3a, lower panel). Scale bar 10 µM. (**b**) Striatal dendrites underwent dendritic beading called “blebs” followed by the regression of the dendritic arborization leading to a phenotype called “pruned”, with few and short dendrites compared to controls. Scale bar 10 µM. (**c**) Quantification of cortical mortality 24 hours post axotomy. (**d**) Quantification of cortical axonal fragmentation 24hours post-axotomy. (**e**) Quantification of striatal pruning, and striatal mortality (**f**) 24 hours post-axotomy. (**g**) Cortical somata and axons pretreated with TTX 5 min before and during the first 5 min after axotomy. After microchannel wash, cultures were further incubated for 24 hours. Scale bar 10 µM. **(h,i)** Quantification of striatal pruning and striatal mortality 24h hours post axotomy after either cortical sham (CTL), or cortical TTX pretreatment.

**Figure 3:**
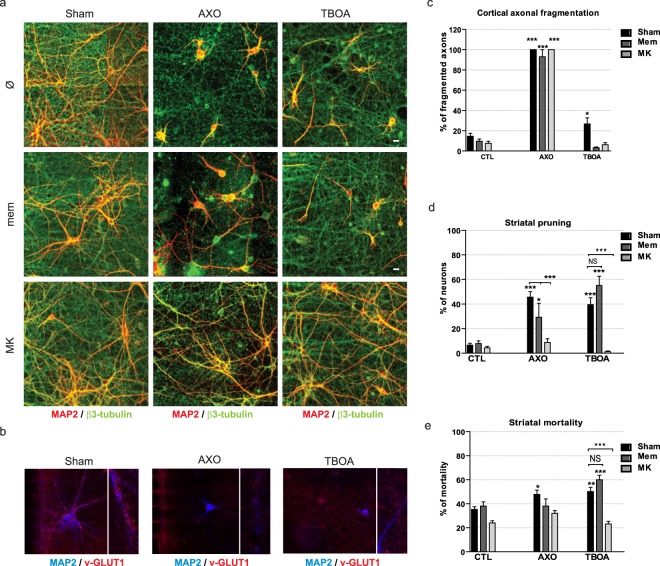
Trans-synaptic degeneration is induced by synaptic NMDAR activation after axotomy or inhibition of glutamate recapture. (**a**) Long-lasting effects of cortical axotomy (AXO) or DL-TBOA treatment combined with pharmacological inhibition of striatal NMDAR. Both cortical axotomy and DL-TBOA altered the survival and the complexity of the dendritic arborization of remaining striatal neurons. Striatal degeneration was inhibited by treatment with 10 µM MK-801 (MK), but not with memantine (Mem; non-competitive low affinity NMDAR antagonist). Cortical axons (α-tubulin staining, green) degenerated after axotomy, but not after DL-TBOA. Scale bar 10 µM. (**b**) Staining of cortical presynaptic VLGUT-1 (red) along striatal dendrites (MAP2, blue) 24h after sham, axotomy, or DL-TBOA treatment. Note that while VGLUT1 staining decreases in the AXO condition, VGLUT1 staining in the DL-TBOA condition is due to VGLUT1 location in intact cortical axons fibers. (**c**) Quantification of cortical axonal degeneration after sham, axotomy or DL-TBOA treatment and striatal NMDAR pharmacological inhibition. Quantification of striatal pruning (**d**) and striatal mortality (**e**) after sham, axotomy or DL-TBOA treatment and striatal NMDAR pharmacological inhibition.

Severing of peripheral or central fibers is widely used as an experimental model of acute trauma, to elucidate the effects of acute deafferentiation on post-synaptic neurons. To assess the impact of axonal injury on distant post-synaptic neurons, we used a microfluidic platform with 3 separate sub-compartments (e.g. proximal, central, and distal), allowing for the manipulation of reconstructed neuronal networks (Fig. 1 and see^23^). Chemical axotomy was performed on the mid portion of cortical axons connected to striatal neurons by perfusing the central compartment with a cell culture medium containing 0.1% Triton for 30 seconds (Fig. 2a « central » panel). As shown in Fig. 2a, severing of cortical axons within the central channels did not damage the proximal fibers within the micro-channels. Indeed, the cortical somato-dendritic compartment did not show any sign of tubulin architecture modification or alteration in cell survival, even 24 hours after axotomy (MAP-2 in red and α-tubulin in green; Fig. 2a,c). Cortical axons in the striatal chamber (Fig. 2a “striatal” panels) displayed no signs of axonal tubulin fragmentation 3 hours after axotomy (middle panel; compare to control condition, upper panel), whereas they were completely fragmented 24 hours after the injury (Fig. 2a; “striatal” panel, Fig. 2d). The architecture of the striatal dendritic tree remained globally unaffected 3 hours post-lesion (MAP-2 labeling in red, Fig. 2a “striatal” panel). Twenty-four hours after the cortical axotomy, both the survival (Fig. 2f), and the complexity of the dendritic arborization of remaining striatal neurons were profoundly altered (Fig. 2a,e). Kinetic analysis showed that striatal dendrites underwent “blebbing” (Fig. 2b “blebs” panel), followed by regression of dendritic arborization, leading to a phenotype called “pruned”, with few and short dendrites compared to controls (Fig. 2a “striatal” and 2b “pruned” panel, quantification in Fig. 2e). We next tested whether axotomy-induced trans-synaptic degeneration could be due to cortical over-activity. To do so, we pre-treated cortical somata and axons with TTX 5 min before performing the axotomy, and TTX was removed 5 min after the axotomy. Cultures were fixed 24 hours later. We found that acute treatment did not protect distal cortical axons from axotomy-induced fragmentation, but fully protected striatal neurons, as revealed by the absence of pruned or dead neurons (Fig. 2g–i). This suggests that dysregulation of cortical activity (in particular over-activity), rather than axonal degeneration *per se* or loss of activity, triggers trans-synaptic degeneration. We next used the non-ratiometric calcium probe Fluo4 to monitor calcium signals in striatal cells upon axotomy of cortical neurons, or striatal blockade of glutamate re-uptake with DL-TBOA. While connected striatal cells exhibit a clear oscillatory pattern synchronous with cortical activity at 0.23 Hz (see^22^ and Supplementary Fig. S1), cortical axotomy and DL-TBOA led to the suppression of striatal rhythmic activity that was replaced by abnormal, long-lasting calcium waves in connected striatal neurons (Supplementary Fig. S1). Twenty-four hours post injury, axotomy alone resulted in complete cortical axon degeneration (Fig. 3c), while axotomy and DL-TBOA treatment led to a decrease in VGLUT1 clustering along striatal MAP2 dendrites, (Fig. 3b), and striatal dendritic pruning (Fig. 3a,d). This was mirrored by a slight increase in striatal neuron death (Fig. 3e).

Extrasynaptic NMDARs, preferentially bearing the GluN2B subunit, have been associated with the recruitment of pro-death signaling pathways^24,25^. We therefore determined whether glutamate-induced trans-synaptic pruning was linked to the initiation of extrasynaptic currents in striatal neurons. To our surprise, memantine, a low affinity non-competitive pan-NMDAR antagonist preferentially targeting extrasynaptic NMDAR subunits^26^, showed only a slight protective effect on striatal pruning induced by axotomy, and no protective effect after striatal DL-TBOA application (Fig. 3a,d). Striatal pruning and mortality was fully blocked by prior incubation with the non-competitive inhibitor MK-801 (Fig. 3a,d,e). These data suggest that axotomy of presynaptic inputs lead to terminal depolarization and glutamate overstimulation in striatal neurons, and finally, to trans-synaptic excitotoxicity. In conclusion, as previously described in brain slices, we show that axotomy of cortical axons leads to trans-neuronal excitotoxicity, and propose a role for synaptic striatal NMDARs in mediating trans-synaptic striatal demise upon cortical axonal insult.

### Chronic alteration of spontaneous cortical rhythms provokes pruning of postsynaptic neurons through GluN2B-containing NMDARs at the cortico-striatal junction

**Figure 4:**
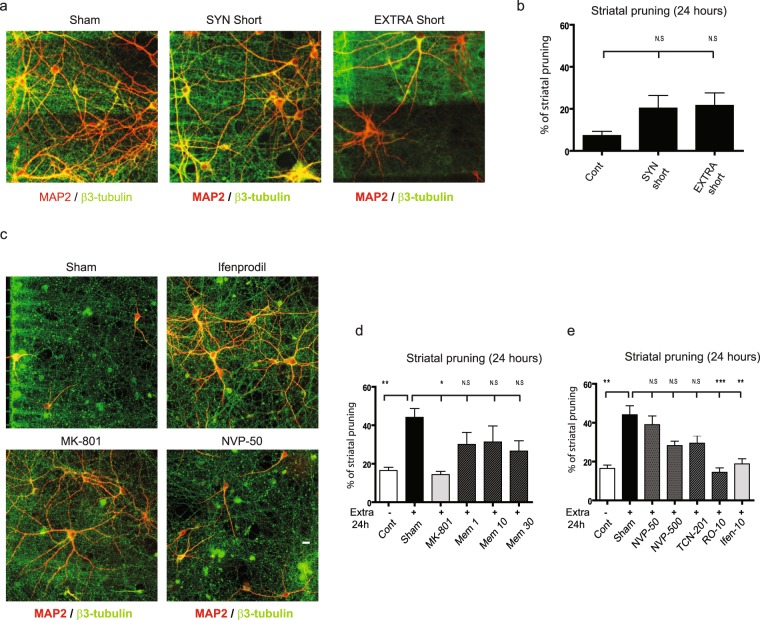
Synaptic and extra-synaptic cortical stimulations induce different trans-synaptic striatal responses. (**a**) Effect of cortical short term synaptic or extra-synaptic treatment on connected striatal neurons. 24 hours after short term cortical synaptic stimulation, striatal dendrites (MAP2, red) and cortical axons (α-tubulin, green) remained unaltered. Slight cortical dendritic beading can be noticed upon extra-synaptic stimulation. Scale bar 10 µM. (**b**) Quantification 24 hours after a short term synaptic, as well as extra-synaptic stimulation shows only minimal effect on striatal pruning. (**c**) Effect of long-term cortical extra-synaptic stimulation on connected striatal neurons after 24 hours shows major signs of pruning of striatal dendrites (MAP2 red) (Sham). Treatment of the striatal chamber with either Ifenprodil or MK-801 reversed striatal pruning, whereas and NVP-50 did not. Note that cortical axons (α-tubulin in green) remained unaltered. Scale bar 10 µM. (**d**) Quantification of striatal pruning 24 hours after long term extra-synaptic treatment (Sham), and reversal by pharmacological inhibitors (MK-80110 µM, Memantine (1, 10, 30 µM). (**e**) Quantification of striatal pruning 24 hours after long term extra-synaptic treatment (Sham), and reversal by pharmacological inhibitors (NVP 50 nM, NVP 500 nM, TCN-201 10 µM, Ro 10 µM, Ifenprodil 10 µM, MK-801 1 µM). One Way ANOVA P < 0.0001, F = 13.56, followed by a post-hoc Bonferroni test (*p-value <0.05; **p-value <0.01; ***p–value <0.001).

While axonal trauma clearly triggers remote neuronal dysfunction, we wondered whether more subtle dysfunctions of cortical axons could trigger a similar spreading of degenerative phenomena. To this aim, we acutely or chronically alterated neuronal rhythms in the cortical chamber, and studied the impact on striatal survival. It is well established that cortical cultures display spontaneous calcium oscillations in basal conditions^27^ that are transferred to striatal neurons in microfluidic networks^22^. As shown in representative calcium traces in Supplementary Fig. S1, short term (10 min) cortical infusion with a Bicuculline/4AP/Nimodipine cocktail preferentially stimulated synaptic NMDAR^28^, and led to an increase in cortical oscillation frequency that translated into a synchronous increase of striatal frequency (approximately 0.23 Hz in both neuronal type). This was not associated with an alteration of striatal survival and pruning (Fig. 4a,b “SYN”). We next altered cortical rhythms by stimulating extra-synaptic receptors^28–30^. Cortical neurons were first treated with Bicuculline and MK-801 for 3 min (Bic+MK), leading to an irreversible block of cortical synaptic NMDAR. Cells were then further exposed to 20 µM glutamate for 3 more min in order to promote non-synaptic NMDAR stimulation. This abolished the synchronous rhythmic pattern of both cortical and striatal neurons with rhythmicity dropping from 0.23 Hz to 0.066 Hz for cortical neurons and 0 Hz for striatal neurons (Supplementary Fig. S1). Interestingly, striatal pruning was not significantly modified after 24 hours (Fig. 4a,b “EXTRA”). This suggests that short-term modification of cortical rhythms does not translate into spontaneous death of striatal neurons. Next, we evaluated the impact of long-lasting alterations of the cortical activity pattern on target striatal neurons by stimulating cortical extra-synaptic NMDARs for 24h. This long-term treatment (reminiscent of mild chronic excitotoxicity) triggered only a modest cortical alteration with few signs of axonal degeneration (data not shown). Conversely, this treatment was associated with trans-synaptic degeneration of striatal neurons, as evidenced by dramatically increased dendritic pruning of striatal neurons (Fig. 4c,d “Sham”). Striatal inhibition of NMDARs by MK-801 completely reversed striatal pruning, suggesting that chronic glutamate dysfunctions trigger a trans-synaptic degeneration mediated by a NMDAR-dependent mechanism (Fig. 4c,d “MK-801”). As previously observed for cortical axotomy and DL-TBOA striatal infusion (Fig. 3), memantine did not confer any protection against abnormal rhythm-induced striatal pruning, even at high (30 µM) doses (Fig. 4d). While not formally excluding extra-synaptic signaling, this indicates that glutamate overspill in the striatal cell culture medium overcomes memantine’s competitive inhibitory effect.

The GluN2B subunit has been proposed to mediate pro-death pathways in neurons, while GluN2A is associated with pro-survival signaling^24,25^. We therefore analyzed which NMDAR subtype was responsible for glutamate-induced trans-synaptic pruning in striatal neurons. Blockade of GluN2A-containing NMDARs with NVP (50 nM or 500 nM), or with 10 µM TCN-201 showed no protective effect, while inhibition of striatal GluN2B-containing NMDARs with 10 µM of Ifenprodil, or 10 µM RO-256981 completely reversed the deleterious effect (Fig. 4e). These results suggest that abnormal glutamatergic transmission following cortical extrasynaptic NMDAR stimulation leads to progressive GluN2B-NMDAR-mediated trans-synaptic pruning of distant striatal neurons.

### Cortical ischemia triggers trans-synaptic neuronal pruning through GluN2B-containing NMDARs at the cortico-striatal junction

**Figure 5:**
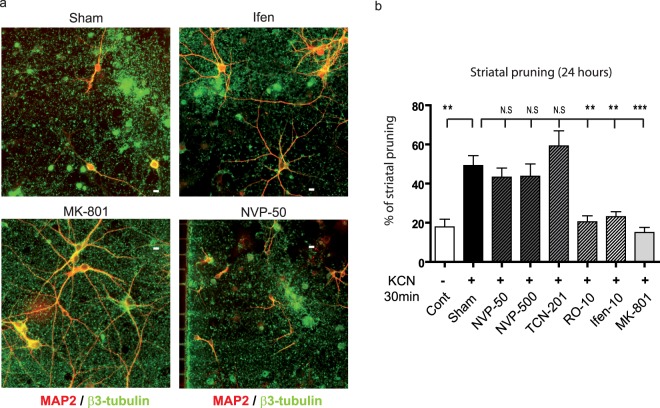
Cortical chemical ischemia induces GluN2B-mediated trans-synaptic striatal dysfunction. (**a**) Effect of short term exposure of cortical neurons to KCN on striatal neurons. 24 hours after short term cortical KCN exposure, striatal dendrites (MAP2 red) showed major signs of pruning (Sham). Treatment of striatal chamber with 10 µM Ifenprodil or MK-801 reversed striatal pruning, whereas 50 nM NVP did not. Note that cortical axons (α-tubulin in green) showed major signs of degeneration in all conditions. Scale bar 10 µM. (**b**) Quantification of striatal pruning 24 hours after KCN exposure (Sham) and reversal by pharmacological inhibitors (NVP 50 nM, NVP 500 nM, TCN-201 10 µM, Ro 10 µM, Ifenprodil 10 µM, MK-801 1 µM). One way ANOVA P <0.0006, F = 8.120, followed by a post-hoc Bonferroni test (*p-value <0.05; **p-value <0.01; ***p–value <0.001).

To gain pathophysiological perspective, we wondered whether mimicking focal ischemia on cortical neurons would trigger a similar scenario in connected striatal neurons. To mimic a focal stroke in our compartmentalized microfluidic platforms, we used a “chemical-ischemia” paradigm^31^ that allows specific treatment of the cortical chamber with 1 mM KCN for 20 minutes. Twenty-four hours after KCN exposure, cortical neurons were degenerated in the cortical chamber, resulting in extensive cortical axon degeneration (data not shown), and striatal pruning in the distant striatal chamber (Fig. 5a,b “Sham”). Inhibition of NMDARs by MK-801 completely reversed striatal pruning (Fig. 5a,b “MK-801”). Reversal of striatal pruning was also observed with inhibition of GluN2B-containing NMDARs with 10 µM of Ifenprodil or 10 µM Ro-256981, whereas blocking of GluN2A-containing NMDARs with either 50 nM NVP, or 10 µM TCN-201, had no significant effect (Fig. 5a,b). These results suggest that abnormal glutamatergic transmission, triggered as a consequence of cortical ischemia leads to progressive GluN2B-NMDAR-mediated trans-synaptic pruning of distant striatal neurons.

## Discussion

Diaschisis-like processes have been classically observed using functional *in vivo* imaging of rodent brain slices that preserve neuro-anatomical pathways^1,3^. This system allows for the study of acute axotomy on distally connected neurons^20,21^; however, adult brain slices only survive for a few hours upon preparation, and are not well suited for studing more chronic processes. Moreover, focal insults such as ischemia or toxin exposure are not well targeted to specific areas on brain slices. Thus, the main purpose of our study was to assess the ability of a variety of cortical insults to trigger trans-synaptic degeneration processes in second order striatal neurons. Using our previously developed microfluidic platforms that allow manipulating 3 separate sub-compartments of oriented neuronal networks^19,22,23^, we demonstrate that both acute and chronic alteration of cortical neurons leads to impairment of striatal survival through abnormal glutamate signaling at the cortico-striatal synapse. Moreover, we found that chronic alteration of cortical rhythms triggered by local stimulation of extra-synaptic NMDARs also leads to trans-synaptic striatal neuron pruning through a GluN2B-NMDAR-mediated process. These results may be regarded as a model for diaschisis-like processes along neuronal pathways in the brain.

In our model, both cortical axotomy and inhibition of glutamate recapture in striatal chambers led to NMDAR-dependent trans-synaptic dysfunction, that was associated with immediate alteration of striatal calcium influx. This shows that we are able to recapitulate in microfluidic networks what had been previously shown using cortico-hippocampal brain slices^32,33^, and is in line with the observation that stretch injury promotes excitotoxic cortical death^34,35^. As axotomy and pro-degenerative insults lead to early synaptic collapse^19,23^, it was conceivable that trans-synaptic alteration could be simply due to passive loss of activity-dependent pro-survival signaling cascades^7^. Yet, long-term blockage of cortical activity with TTX did not lead to spontaneous striatal degeneration or regression. Although this does not rule out the possibility that silencing cortical activity for longer time periods (>48h) may trigger spontaneous cortical and striatal degeneration, or increase vulnerability to intercurrent insults^10,36^, our results indicate that abnormal cortical activity, rather than passive loss of synaptic contacts, is responsible for trans-synaptic striatal degeneration after focal insults. Cortical synaptic stimulation induced an increase in calcium burst frequency in both cortical and connected striatal neurons, and did not trigger any deleterious effect on striatal survival. Despite this, we did observe a slight decrease in VGLUT1 clustering at the cortico-striatal junction upon chronic Bicuculline/4AP/Nimodipine treatment. These observations could be consistent with data showing that a transient increase of burst frequency is associated with protective effects^11,37,38^, whereas long-term epileptic patterns initiate degenerative processes^39,40^. Alternatively, they may be indicative of plasticity-dependent events occurring at the cortico-striatal synapse upon long term modification of excitatory innervation^41^. Pruning of striatal dendrites was triggered to the same extent by all different cortical insults, including axotomy, ischemia, and mild excitotoxicity. However, while both axotomy and KCN led to axonal degeneration and elimination of cortical pre-synaptic structures, the latter were not significantly altered by DL-TBOA and cortical extra-synaptic signaling, which provoke progressive striatal pruning, leaving cortical terminals intact. Taken together, our observations strongly suggest that presynaptic regulation of glutamate neurotransmission is a central gatekeeper of striatal survival in cortico-striatal networks. Not only do our results confirm the concept of diaschisis at the cellular level, we also demonstrate that sub-toxic glutamatergic insults at the cortical level are sufficient to trigger distant dysfunction in second order neurons.

Many studies have shown an association of excitotoxic neuronal death with extra-synaptic NMDA currents during neurodegenerative processes provoked by a variety of insults such as stroke and epilepsy^7,25,28,36,42,43^. Accordingly, we were surprised to find that memantine, even at high doses, failed to protect striatal neurons in our assay. In contrast, we consistently observed that MK-801, which antagonizes only activated NMDARs, prevented striatal trans-synaptic pruning. As memantine is thought to inhibit extra-synaptic receptors rather specifically, MK-801 inhibits all activated NMDARs regardless of their localization (synaptic or extra-synaptic), suggesting that degeneration after cortical insult would be due to over-activation of synaptic receptors. Our results are in agreement with studies showing that synaptic NMDARs of hippocampal neurons can directly mediate excitotoxicity when the source of glutamate arises from synapses^40^. Furthermore, specific degradation of glycine, a co-agonist of extra-synaptic receptors, does not protect against NMDA-induced excitotoxicity, whereas degradation of D-serine, a co-agonist of synaptic NMDARs, exhibits a protective effect^44^. Despite these findings, we cannot exclude that the high glutamate concentrations released by massive cortical innervation in the striatal chamber may counteract memantine’s competitive effects. An alternative explanation would be that NMDARs are not specifically localized at extra-synaptic sites in striatal neurons under basal conditions; but that chronic degenerative processes may enhance the routing of NMDARs to extra-synaptic sites. Indeed, mutant Huntingtin has been shown to enhance the extra-synaptic localization of NMDARs^16,45^. It is well-known that synaptic and extra-synaptic NMDARs are composed of a combination of NMDAR subunits. Accordingly, our pharmacological assessment with specific inhibitors suggested that the GluN2B subunit is primarily involved in striatal trans-synaptic dysfunction in our culture conditions. Both NVP*-*AAM077 and TCN-201, which preferentially inhibit NMDAR GluN2A subunits, showed no protective activity, whereas Ifenprodil and Ro-256981 preferentially inhibit GluN2B subunits, resulting in the reversal of striatal pruning. This is consistent with recent reports of GluN2B mediating excitotoxic signaling in neurons^46^. In conclusion, our results demonstrate that an acute axonal trauma, somatic ischemia-related modification of cortical physiology, or chronic alteration of cortical firing rhythms can trigger a distant and excitotoxic striatal neuron dysfunction reminiscent of diaschisis^1^. Furthermore, they confirm once again that microfluidic platforms permit the reconstruction of neuronal pathways *in vitro*, and are valuable tools to study neurodegenerative syndromes at the network level.

## Methods

### Master fabrication and microfluidic chip production

The microfluidic channels are composed of large channels (55 µm in height) for cell injection and thin channels (3 µm in height) for axon growth. To fabricate the template, we used two layers of photoresist (SU82005 and SY355). Silicon templates were replicated within another polyester resist (Dalbe) as previously described (these two processes were previously detailed^22,47^). The three compartment chips (“3C”) are composed of two rectangular macro-channels (length: 4000 µm; width: 500 µm; height: 55 µm) separated by arrays of asymmetrical micro-channels (length 500 µm, 15 to 3 µm width, 3 µm height), interrupted by a third macro-channel inserted in the middle of the micro-channel “diode” array (as previously described^48^). This third macro-channel acts as an intermediate compartment allowing control of the flow of solution over the mid-portion of cortical axons^48^. Briefly, the chips were produced for cell culture by mixing PDMS (Sylgard 184) with curing agent (9:1 ratio), and degassing under vacuum. The resulting preparation was poured into a polyester resin replicate and reticulated at 70°C for 2 hours. The elastomeric polymer print was detached and 2 reservoirs were punched for each macro-channel. The resulting piece was cleaned with isopropanol and dried. The polymer print and a glass cover slip were treated for 200 sec in an air plasma generator (98% power, Diener ATTO) and bonded together. Chips were placed under UV for 15 minutes and then coated with a solution of poly-D-lysine (10 µg/mL) overnight, and washed with PBS before cell seeding.

### Primary neuronal cultures

Swiss mice were purchased from René Janvier (Le Genest Saint Isle, France) and cared for by the animal care facility at University Pierre et Marie Curie (IFR83). Animal care was conducted in accordance with standard ethical guidelines (U.S. National Institutes of Health publication no. 85–24, revised 1985, and the European Committee Guidelines on the Care and Use of Laboratory Animals), and the local IBPS and UPMC ethics committee approved the experiments (in accordance with the standard ethical guidelines of the CNRS “Formation à l’Expérimentation Animale” and were approved by the “C2EA - 05 Comité d’éthique en experimentation animale Charles Darwin”). Cortices and striata were micro-dissected from E14 embryos of Swiss mice (René Janvier, France). All steps of dissection were performed in cold PBS supplemented with 0.1% glucose. Dissected areas were digested with trypsin-EDTA for striata (Gibco) or papaïn for cortices (20 U/mL in DMEM, Sigma). Following tryspin or papaïn inactivation with FBS, the tissue was mechanically dissociated with a pipette in presence of DNAse. After several rounds of rinsing, cells were re-suspended in DMEM, to a final density of 45 million cells/mL for cortices and 12 million/mL for striata. Cortical cells were then seeded in the somatic compartment and striatal cells in the distal compartment: 3 µL of the cell suspension was introduced into the upper reservoir and cells flowed into the chamber and adhered within 1–2 min. Cell culture medium was then added equally to the four reservoirs (60 µL/reservoir). Both neuronal cell types were grown in DMEM glutamax + streptomycin/penicillin (Gibco) + 10% FBS + 1% N2 (Gibco) + 2% B27 (Gibco). Microfluidic chips were placed in plastic Petri dishes containing H_2_O-EDTA to prevent evaporation, and incubated at 37°C in a humid 5% CO_2_ atmosphere. The culture medium was renewed every 6 days. Typically, after 15 days *in vitro*, cortical neuronal cultures were composed of 90% neurons and 10% astrocytes, while striatal cultures were composed of 80% neurons and 20% astrocytes.

### Pharmacological treatment and manipulation of neuronal networks

For pharmacological pretreatments, medium was removed from the striatal chambers, and was replaced by complete cell culture medium containing the pharmacological drugs. The following concentrations were used: memantine (1 µM, sigma), MK-801 (10 µM, sigma), Ifenprodil (α-(4-hydroxyphenyl)-β-methyl-4-benzyl-1-piperidineethanol (+)-tartrate salt, 10 µM; Sigma). For pharmacological treatments, drugs were perfused to the striatal chamber 5–15 min before treatments and kept for the entire duration of the experiment.

### Glutamate recapture inhibition

Glutamate recapture inhibition was performed by flowing DL-threo-β-benzyloxyaspartate (DL-TBOA, 30 µM, Tocris) to the striatal chamber to inhibit the glutamate transporters (EEATs) on neurons and glial cells for 24 hours.

### Cortical activity blockade

Cortical activity was blocked by flowing tetrodotoxine (TTX, 1 µM, Tocris) within the somato-dendritic (proximal chamber) and mid-axon (central chamber) compartments of cortical neurons, whereas the distal chamber was not treated. Short TTX treatment was applied for 10 min in control conditions. For the axotomy paradigm, TTX was applied 5 min before axotomy, and remained in contact with cells for 5 min following axotomy, after which the chambers were washed with fresh medium.

### Synaptic and Extra-synaptic stimulation

Based on previously published synaptic stimulation protocols^28–30^, the following cocktail was applied to the somato-dendritic cortical chambers for 10 min: Bicuculline (Bic, antagonist of GABAergic neurons, 50 µM, Tocris); 4-Aminopyridine (4-AP, antagonist of potassium channels type-A to weakly depolarize membrane and facilitate action potentials, 2.5 mM, Tocris); Nimodipine (Nimo, antagonist of voltage-dependent dihydropyridine calcium channels to block calcium influx through other channels than ionotropic glutamate receptors, 5 µM, Tocris). Following this stimulation cocktail, the somato-dendritic cortical chambers were then washed with conditioned medium. For the abnormal synaptic transmission experiment, a previously described 3-step protocol was used for extrasynaptic cortical stimulation within the somato-dendritic cortical compartments. Step one consists of the activation and almost simultaneous blockade of synaptic receptors by perfusing Bicuculline (50 µM, Tocris) with MK-801 (10 µM, Tocris) for 3 min. Step two is a rapid wash of the previously applied drugs. Step three is the activation of extrasynaptic receptors with glutamate (20 µM, sigma) for 3 min. For long term treatment, step 3 was allowed to proceed for 1 hour. Cortical and striatal chambers were then washed with complete medium with or without pharmacological inhibitors, and returned to a 37°C, 5% CO_2_ incubator for 24 hours before fixation.

### Chemical hypoxia

Cortical chemical hypoxia was triggered by introducing 1 mM Potassium Cyanide (KCN, Sigma Aldrich) to the cortical chamber for 20 minutes. Concomitant pharmacological blockade of striatal neurons was performed by co-flowing the pharmacological inhibitors into the striatal chamber. Cortical and striatal chambers were then washed with complete medium with or without pharmacological inhibitors and returned to the 37°C, 5% CO_2_ incubator for 24 hours before fixation.

### Neuronal network axotomy

After full maturation of the neuronal network (14 days after seeding), cell culture medium was removed from the two reservoirs connecting the central chamber and a solution of plain DMEM (sham) or 0.1% Triton in DMEM was flowed from the upper reservoir for 30 sec. Careful attention was paid to the fluidic isolation of the central channel: the two outer cell culture chambers were pressurized with 50 µL of cell culture medium while 20 µL of sham or detergent-containing medium was introduced into the upper reservoir of the central channel. This created a flux of detergent in the central chamber containing cortical axons. The central chamber was then washed three times with DMEM and the central reservoirs were then filled with medium. Axotomized cells were then kept at 37°C under 5% CO_2_ for various periods before fixation.

### Immunofluorescence

Cultures were fixed in 4% paraformaldehyde (PFA) for 20 min at room temperature (RT). Cells were then washed twice with PBS for 5 min and permeabilized for 45 min with 0.2% Triton X-100 and 1% BSA in PBS. Primary antibodies were then added and the samples were incubated at 4°C overnight in PBS. The samples were rinsed twice for 5 minutes with PBS and further incubated with the corresponding secondary antibodies for 2 hours at RT. The chips were then rinsed once with PBS and once with PBS + 0.1% sodium-azide, and mounted in moviol mounting medium. The following primary antibodies were used: α-tubulin-FITC (mouse monoclonal 1/700, Sigma); MAP-2 (mouse monoclonal 1/500, Sigma); VGLUT1 (rabbit polyclonal 1/500, gift E. Herzog, CNRS, UMR 5297). Species-specific secondary antibodies coupled to Alexa 350, 488, and 555 were used (1/500, Invitrogen) to visualize bound primary antibodies.

### Image acquisition

Images were acquired with an Axio-observer Z1 (Zeiss) fitted with a cooled CCD camera (CoolsnapHQ2, Ropert Scientific). The microscope was controlled with Metamorph software and images were analyzed using ImageJ software and self-developed Image J macro analyses.

### Quantification of dendritic pruning, synaptic disconnection and striatal death

Striatal death was assessed by counting the number of neuronal condensed nuclei after staining with Hoechst and MAP2 antibody. Dendritic pruning was assessed after MAP2 staining. Connected healthy striatal neurons after 15 DIV classically exhibit 4 to 6 primary dendrites subdivided in secondary and tertiary dendrites. Striatal neurons were considered as pruned when they were devoid of secondary and tertiary dendrites with reduced primary dendrites (<4). Synaptic loss was assessed by counting VGLUT1 clusters in the entire image. Images were all obtained with the same acquisition parameters. The images were similarly processed with ImageJ software before being used for quantification: the brightness/contrast of all control images was optimized manually to eliminate the background and to maximize the signal. The means of the minimum and maximum intensities were then calculated in the control condition. We applied these same settings to all images. As VGLUT1 is specifically expressed in cortical neurons and not striatal neurons, VGLUT positivity was used as a readout to quantify cortical pre-synaptic elements docked on striatal dendrites, as previously shown^22,23^. The images of the three stainings VGLUT1/α-Tubulin/MAP-2 were merged, and the resulting image was used to define images with homogenous innervations of striatal neurons by cortical fibers. The MAP2 and VGLUT1 images were then used for automated quantification using image J software (plugin spot detector).

### Calcium Imaging analysis

After 14 days *in vitro*, micro-chambers were rinsed twice with loading buffer (NaCl 129 mM, KCl 4 mM, MgCl_2_ 1 mM, CaCl_2_ 2 mM, Glucose 10 mM, Hepes 10 mM dissolved in sterile water). Cells were then incubated for 30 min at RT, with 2 mM fluo4 calcium probe (Invitrogen) dissolved in loading buffer. After 2 rinses, cells were returned to complete cell culture medium and recorded, at RT, under the microscope (see above). Each frame was acquired every 500 ms for 5 minutes. Calcium trace analysis was performed using the Delta F/F0 plugin (WCIF image J plugin) of Image J software. Corrected fluorescence intensity oscillations of individual cells were plotted with version 5 of GraphPad Prism® software.

### Statistical analysis

Statistical analyses for neuronal pruning were performed with the program StatView 5® or Graph Pad Prism®. All variables were initially analyzed with the Kolmogorov–Smirnov test to determine whether data varied significantly from the pattern expected, and if the data was drawn from a population with a normal distribution. Variables that passed the normality test were analyzed by one-way ANOVA (treatment) for Paired Data (pruning) followed by Fisher PLSD post-hoc tests (p < 0.05). For the VGLUT1/MAP2 colocalization, differences were assessed by ANOVA with Bonferroni post-hoc tests, where appropriate, and data were presented as mean ± S.E.M. For all analyses: *p-value <0.05; **p-value <0.01; ***p–value <0.001. Provided statistical data are the results of at least 3 independent experiments conducted in triplicate.

## Electronic supplementary material

Supplementary Figure 1
