# Supplementary material for: Dysregulated Neurotransmission induces Trans-synaptic degeneration in reconstructed Neuronal Networks
Source: Sci Rep. 2018 Aug 2;8:11596. doi: 10.1038/s41598-018-29918-1 (PMC6072786; doi:10.1038/s41598-018-29918-1)
Supplement: Supplementary file 1 — Supplementary Figure 1 [file 41598_2018_29918_MOESM1_ESM.pdf]

Dysregulated Neurotransmission induces Trans-synaptic degeneration in reconstructed Neuronal Networks.

Authors and affiliations: Bérangère Deleglise [1,2*], Benjamin Lassus [1,2*], Vanessa Soubeyre [1,2], Mohamed Doulazmi [1,2], Bernard Brugg [1,2], Peter Vanhoutte [3] and Jean-Michel Peyrin [1,2+]

[1] CNRS UMR 8256, Biological Adaptation and Ageing, Paris, F-75005, France

[2] Sorbonne Universités, UPMC Université Paris 06, CNRS/UMR 8256, B2A, Biological Adaptation and Ageing, Institut de Biologie Paris Seine, Paris, F-75005, France

[3] Sorbonne Universités, UPMC Université Paris 06, CNRS/UMR8246-INSERM/UMR-S1130, Institut de Biologie Paris Seine, Paris, F-75005, France

[*]Sharing Co-Authorship

Supplementary information

Supplementary FIGURE S1 Legend: Cortical chemical ischemia induces GluN2B-mediated trans-synaptic striatal dysfunction.

For synaptic stimulation (SYN), cortical neurons were treated with bicuculline, 4-AP, and Nimodipine for 10 min. For extrasynaptic stimulation (EXTRA) cortical neurons were treated first with bicuculline and MK-801 for 3 min (Bic+MK), washed for 2 min, and then further incubated with glutamate for 3 min (Glut). **a.** Fluo-4 non-ratiometric calcium recording of connected cortical (upper lane) and striatal (lower lanes) neurons after either synaptic (left panels) or extra-synaptic (right panels) stimulation. Left Panels: brief stimulation of cortical synaptic NMDAR induced an increase of synchronous calcium oscillation in both cortical and striatal neurons. Cortical axotomy abolished striatal oscillatory rhythms and induced an immediate and brief calcium influx in striatal neurons, followed by a secondary slow wave of calcium influx (representative trace of Fluo4 non-ratiometric calcium analysis from striatal chambers). Striatal incubation with DL-TBOA (30 μM) abolished striatal rhythms and induced progressive calcium influx in striatal neurons.

b. Brief extra-synaptic NMDAR cortical stimulation (synaptic inhibition with bicuculline and MK-801 followed by extra-synaptic activation by glutamate) induced slow and weak, asynchronous cortical calcium oscillations that did not induce striatum calcium influx during the stimulation time. Representative calcium traces of six individual neurons per condition.

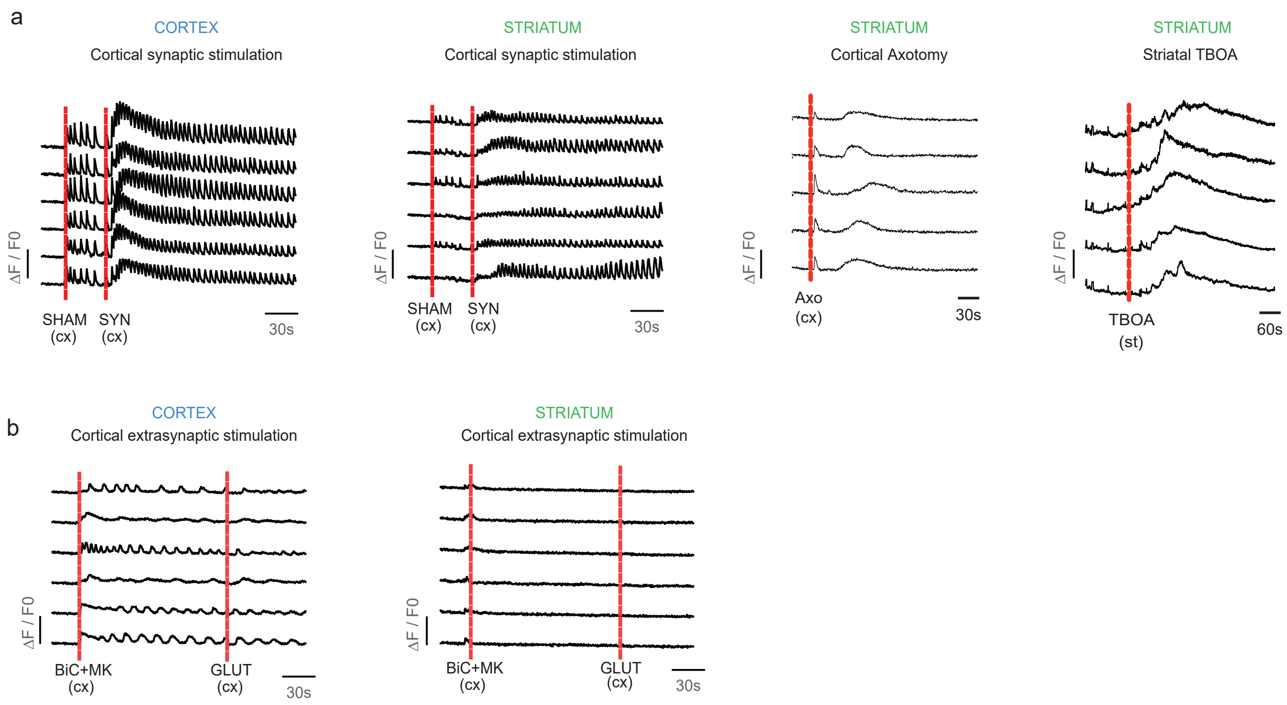

a
CORTEX
Cortical synaptic stimulation
ΔF / F0
SHAM
(cx)
SYN
(cx)
30s
STRIATUM
Cortical synaptic stimulation
ΔF / F0
SHAM
(cx)
SYN
(cx)
30s
STRIATUM
Cortical Axotomy
ΔF / F0
Axo
(cx)
30s
STRIATUM
Striatal TBOA
ΔF / F0
TBOA
(st)
60s
b
CORTEX
Cortical extrasynaptic stimulation
ΔF / F0
BiC+MK
(cx)
GLUT
(cx)
30s
STRIATUM
Cortical extrasynaptic stimulation
ΔF / F0
BiC+MK
(cx)
GLUT
(cx)
30s
